# 83678 Bridging Gaps to Equalize Community-Academic Partnership: A Comparison of Capacities With Research Needs Across CTSA Program Hubs

**DOI:** 10.1017/cts.2021.683

**Published:** 2021-03-30

**Authors:** Bonnie Spring, David Moskowitz, Angela F. Pfammatter, Ruchi Patel, Hannah Rumsey

**Affiliations:** Northwestern University Feinberg School of Medicine

## Abstract

ABSTRACT IMPACT: Our research identifies key opportunities for increased cross-CTSA collaboration, as a means to improve community-research cooperation and better CBPR practices. OBJECTIVES/GOALS: Currently, team science training prioritizes developing the collaborative competencies of interdisciplinary scientists to work with each other and, more recently, with communities. Community-facing team science resources are scarce but present among some CTSAs, suggesting that capacity gaps might be remedied through cross-hub collaboration. METHODS/STUDY POPULATION: We reviewed online information provided by the 62 current CTSAs to identify: (1) which hubs engage in community research, and (2) what resources the hubs utilize to orient, train, and support community stakeholders as research partners. We then examined the capacities of the collectively available CTSA resources to address needed knowledge, skills, and attitudes that community-engaged researchers have identified as essential for community-based stakeholders to partner equally in research. Finally, we explored practical challenges in team-based dynamics (e.g., interpersonal difficulties, expertise gaps, resource management) that may facilitate or hinder communities’ research endeavors, and suggest resources that CTSAs might implement to facilitate team science dynamics. RESULTS/ANTICIPATED RESULTS: Hubs (n=59) have community engagement programs, 12 of which provide community-based participatory research toolkits. Toolkits vary from basic checklists to fully developed modules. Some hubs also offer consultation services and partner match-making. Learning objectives include: outcome definition, logic models, and goal-setting. Learning resources remain underdeveloped to help communities appreciate the benefits of research engagement and convince academic partners of the value of real-world knowledge and community improvement relative to scientific advancement. Also lacking is easily accessible support to understand the research process, build verifiable trust, maintain bidirectional knowledge and assets, and implement consistent, best practice methodological and reporting protocols. DISCUSSION/SIGNIFICANCE OF FINDINGS: Gaps between current hub offerings and community needs suggest prioritizing creation of resources whose learning objectives highlight the benefits of research engagement for community partners; foster mutual values affirmation between partners; and offer tools that build warranted community-researcher rapport.

